# Defect-Engineering of 2D Dichalcogenide VSe_2_ to Enhance Ammonia Sensing: Acumens from DFT Calculations

**DOI:** 10.3390/bios13020257

**Published:** 2023-02-11

**Authors:** Gopal Sanyal, Surinder Pal Kaur, Chandra Sekhar Rout, Brahmananda Chakraborty

**Affiliations:** 1 Mechanical Metallurgy Division, Bhabha Atomic Research Centre, Trombay, Mumbai 400085, India; 2 Department of Chemistry, Indian Institute of Technology Ropar, Rupnagar 140001, India; 3 Centre for Nano and Material Sciences, Jain Global Campus, Jakkasandra, Ramanagaram, Bangalore 562112, India; 4 High Pressure and Synchroton Radiation Physics Division, Bhabha Atomic Research Centre, Trombay, Mumbai 400085, India; 5 Homi Bhabha National Institute, Mumbai 400094, India

**Keywords:** 2D materials, VSe_2_ monolayer, ammonia sensing, electronic properties, reversible sensors, density functional theory

## Abstract

Opportune sensing of ammonia (NH_3_) gas is industrially important for avoiding hazards. With the advent of nanostructured 2D materials, it is felt vital to miniaturize the detector architecture so as to attain more and more efficacy with simultaneous cost reduction. Adaptation of layered transition metal dichalcogenide as the host may be a potential answer to such challenges. The current study presents a theoretical in-depth analysis regarding improvement in efficient detection of NH_3_ using layered vanadium di-selenide (VSe_2_) with the introduction of point defects. The poor affinity between VSe_2_ and NH_3_ forbids the use of the former in the nano-sensing device’s fabrications. The adsorption and electronic properties of VSe_2_ nanomaterials can be tuned with defect induction, which would modulate the sensing properties. The introduction of Se vacancy to pristine VSe_2_ was found to cause about an eight-fold increase (from −012 eV to −0.97 eV) in adsorption energy. A charge transfer from the N 2p orbital of NH_3_ to the V 3d orbital of VSe_2_ has been observed to cause appreciable NH_3_ detection by VSe_2_. In addition to that, the stability of the best-defected system has been confirmed through molecular dynamics simulation, and the possibility of repeated usability has been analyzed for calculating recovery time. Our theoretical results clearly indicate that Se-vacant layered VSe_2_ can be an efficient NH_3_ sensor if practically produced in the future. The presented results will thus potentially be useful for experimentalists in designing and developing VSe_2_-based NH_3_ sensors.

## 1. Introduction

With the development of technology, the requirement for gas sensors in the fields of industry, agriculture, medicine, air-quality monitoring, etc., has been amplified [1,2]. For instance, gases such as carbon monoxide, nitrogen oxide, nitrogen dioxide, ammonia, etc. are harmful to living beings and can trigger serious health issues [3,4]. To eliminate such hazardous gases from the environment, lucrative sensors with good stability, sensitivity, and selectivity are desirable. In the past, metal oxides such as ZnO, SnO_2_, and so on were explored as efficient sensors having good sensitivity and selectivity towards the sensing of harmful gases [5]. Although metal oxides are cheaper and need low fabrication costs, their elevated operating temperature restricts their use in sensing devices [6]. Following this, various types of sensing materials have been reported in the past. Among all the reported sensing materials, chemi-resistors are recommended as promising sensitive and selective sensors [7,8]. For instance, Oudenhoven et al. reported a thin layer of ionic liquid [BMIM][NTf_2_] as the electrolyte, capable of sensing NH_3_ even at a level of 1 ppm [9]. On the other hand, Amirjani et al. reported a calorimetric sensor for detecting NH_3_ by utilizing localized surface plasmon resonance of Ag nanoparticles for detection in the range of 10–1000 mg L^−1^ [10]. The electrochemical sensor developed by Arya et al. uses SnO_2_ nanoparticles synthesized with the sol–gel route to sense NH_3_ in aqueous solution [11].

Graphene is a two-dimensional carbon allotrope with a zero band gap and possesses a high surface-to-volume ratio [12,13]. The discovery of graphene brought a breakthrough in the exploration of two-dimensional nanomaterials [14,15,16,17,18]. Due to the presence of novel physical and chemical properties, two-dimensional nanomaterials can be used in a wide range of applications such as energy storage devices, catalysis, sensing devices, etc. [19,20,21]. The application of two-dimensional materials, namely borophene, phosphorene, transition metal dichalcogenides (TMDs), etc., in gas sensing has been studied by different research groups [19,20,21,22,23,24,25,26]. For instance, honeycomb germanium is reported to act as an efficient sensor as compared to graphene-based sensors [27,28]. Sosa and his coworkers investigated the application of alkali, alkaline earth metals, and transition metal-doped germanene in ammonia (NH_3_) sensing by computing adsorption energies, charge transfer analysis, work function, and desorption time [29]. Several other studies have also been reported in the past to investigate the adsorption properties of NH_3_ on different two-dimensional materials [30]. For instance, adsorption energies and diffusion energy barriers were computed for NH_3_ adsorption on MoO_3_ nanomaterial by Xu and coworkers [31]. The authors reported the low sensing of NH_3_ on the studied two-dimensional material. Lv and his coworkers studied the sensing properties of NH_3_ on a two-dimensional C_3_N monolayer by performing density functional theory [32].

Transition metal dichalcogenide nanosheet; MoSe_2_ is reported to act as an efficient sensor in the sensing of CO, NO, NO, and NO_2_ gases [33,34]. It is also possible to tune the physical and chemical properties of such two-dimensional nanomaterials by tuning their structures [35,36,37,38,39,40,41,42,43]. The Janus TMDs are two-dimensional nanomaterials in which a metal layer is sandwiched between two different non-metal atom layers. The difference in the non-metal atom layers introduces asymmetry, which is responsible for enhancing the physicochemical properties of such materials. The Janus TMDs have been explored for their use in hydrogen storage, catalysis, water splitting, etc. [44,45,46,47]. Along with these properties, the application of Janus TMDs in gas sensing has also been studied by researchers in the past [48,49,50]. For instance, the role of MoSSe nanomaterial in the sensing of CO, CO_2_, NO, and NO_2_ was studied using DFT methods [46]. The authors reported that the selectivity of sensing can be improved with the help of external strain. Following this, the sensing properties of the defected Janus TMDs have also been studied in the past [51]. The studies showed that the defected Janus TMDs showed higher sensitivity towards the gas molecules as compared to pristine monolayers.

**Figure 1:**
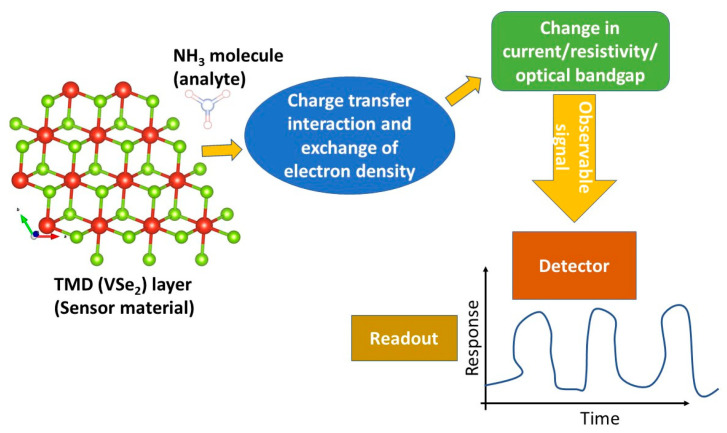
Schematic flow diagram of gas sensing mechanism involving charge transfer interactions.

The charge transfer between adsorbate and adsorbent partakes in the gas sensing mechanism (Figure 1). Previous studies showed that the gas-sensing behavior of two-dimensional can be improved by introducing p-type or n-type doping [52,53,54,55]. The doping can be introduced by incorporating impurities in the two-dimensional nanomaterial lattice [56,57]. Suh and his group reported the hole generation in the MoSe_2_ monolayer with the doping of Nb in the lattice structure [58]. The gas-sensing behavior of Nb-doped MoS_2_ nanosheets has been investigated by Choi and his coworkers [59]. Their report stated that optimum NO_2_ sensing of MoS_2_ can be enhanced up to 8% with Nb doping and hence, can be considered an effective way to achieve high-performance gas sensing devices. The improvement of the gas-sensing behavior of MoSe_2_ and MoTe_2_ nanomaterials with the elemental substitution is also reported in the past [60]. The role of V, Nb, and Ta-doped MoS_2_ in NH_3_, H_2_O, and NO_2_ sensing has been studied by Zhu and his group [61]. Authors suggested that doping of transition metal atoms enriches the sensing properties of MoS_2_. The effect of Al, Si, and P-doped MoS_2_ on the adsorption as well as sensing of NH_3_ has been studied by Luo and his group [62]. The effect of nitrogen, phosphorus, and arsenic doping on the CO, NO, and HF sensing of Janus WSSe nanosheets has been studied in the past using DFT methods [63]. The studies showed that ~3.12% doping of nitrogen, phosphorus, and arsenic makes Janus WSSe nanosheets efficient sensing materials even without imposing external strain. The utilization of VSe_2_ nanomaterial for the sensing of nitrobenzene and catechol has been reported in past studies [64,65]. Vacancy engineering has been reviewed as a critical strategy for tuning electron and phonon structures of two-dimensional materials in general and for gas-sensing applications in particular [66,67,68]. For instance, in the case of TMDs, the introduction of vacancy has been reported to be beneficial for the sensing of SO_2_, NH_3_, NO_2_, ‘NO, O_2_, and CO, and decomposed SF_6_ gases in SnSe_2_, SnS_2_, MoS_2_, PtSe_2_, and WS_2_ layered systems, respectively [69,70,71,72,73]. Keeping the above in mind, the potential of vacancy-engineered VSe_2_ for the detection of NH_3_ appears to be a still unaddressed topic, to the best knowledge of the authors.

The modality of detection of NH_3_ with VSe_2_ nanosheets has thus been theoretically studied in the present work. The effect of defect-engineered nanosheets has also been considered in this work by introducing V-defected as well as Se-defected layered VSe_2_ nanomaterials. Using first-principles calculations, the change in the electronic and magnetic properties of defected VSe_2_ monolayers has been compared with the pristine material. The sensing capabilities of pristine and defected VSe_2_ monolayers have also been assessed in terms of adsorption energy values, electronic, magnetic, and charge transfer properties with the NH_3_ molecule.

## 2. Computational Methods

The density functional theory (DFT) computations were accomplished by means of the Projector Augmented Wave (PAW) principles as implemented in the Vienna ab initio Simulation Package (VASP) [74,75,76,77]. In the simulations, generalized gradient approximation (GGA) was used for exchange-correlation functions [78]. During the computations, the convergence criteria for Hellman–Feynman forces were kept at 0.01 eV/Å alongside the plane wave cut-off energy of 600 eV. The long-range interactions may impact the sensing properties of the material. Hence, long-range interactions were taken care of with Grimme’s DFT-D3 functional [79,80]. The Γ-centered K-points grid of 6 × 6 × 1 was used for the integration of the first Brillouin zone [81]. A vacuum of 20 Å was introduced in the z-direction to avoid the interactions between the layers in the Z direction. The thermal stability of the VSe_2_ monolayer adsorbed with NH_3_ was computed with the help of ab-initio molecular dynamics simulations (AIMD). The AIMD simulations were carried out in the NVT ensemble using the Nosé–Hoover thermostat to determine the thermal stability of VSe_2_ + NH_3_ and VSe_2_(Se_v_) + NH3 systems at 400 K. The simulations were carried out for a total time of 5 ps with a time step of 1 fs.

## 3. Results and Discussion

### 3.1. Structural Analysis of Pristine and Defected VSe_2_

**Figure 2:**
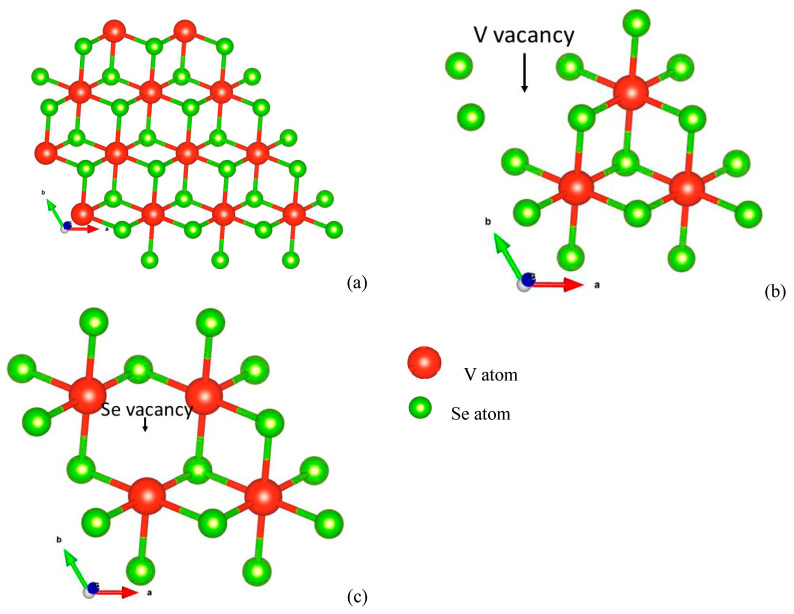
Relaxed structure of (**a**) pristine VSe_2_, (**b**) VSe_2_ deficient with V atom, and (**c**) VSe_2_ deficient with Se atom.

The 4 × 4 × 1 supercell of VSe_2_ was used to mimic the two-dimensional monolayer in this work. The geometry-relaxed structure of pristine VSe_2_ is shown in Figure 2a. In this structure, the metal atom layer is embedded between the selenium atom layers. Using the optimized structure of pristine VSe_2_, V-defected VSe_2_ was constructed by removing a single V-metal atom from the monolayer [Figure 2b]. Similarly, the Se-defected layer was modeled by eliminating a Se-atom from the monolayer [Figure 2c]. The V and Se-defected monolayers are described as VSe_2_(V_v_) and VSe_2_(Se_v_), distinctly. The optimized structures of VSe_2_, VSe_2_(V_v_), and VSe_2_(Se_v_) are used for the further adsorption of the NH_3_ molecule at various possible positions, as mentioned below.

### 3.2. Adsorption of NH_3_ on VSe_2_, VSe_2_(V_v_), and VSe_2_(Se_v_)

**Figure 3:**
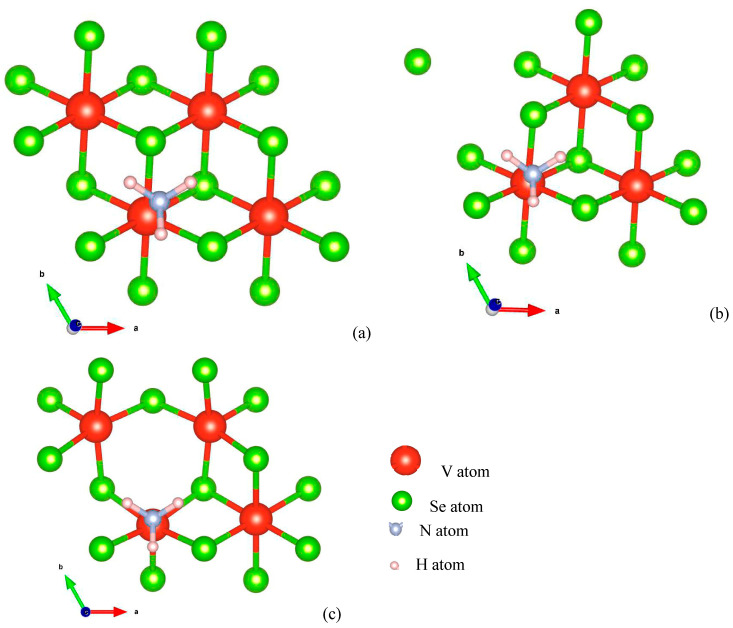
Relaxed structures of (**a**) NH_3_ (N atom directly placed above V atom) on pristine VSe_2_, (**b**) NH_3_ (N atom directly placed above V atom) on VSe_2_ deficient with V atom, and (**c**) NH_3_ (N atom directly placed above V atom) on VSe_2_ deficient with Se atom.

To understand the NH_3_ sensing of pure and defected VSe_2_, the NH_3_ molecule was placed at various possible sites, 2 Å above the VSe_2_, VSe_2_(V_v_), and VSe_2_(Se_v_) monolayers. The structurally relaxed geometries upon NH_3_ introduction on VSe_2_, VSe_2_(V_v_), and VSe_2_(Se_v_) monolayers are depicted in Figure 3. The stability of the NH_3_ adsorbed complexes is assessed in terms of adsorption energy values both with and without van der Waals (VdW) interactions.

The adsorption energy is computed using the following equation:

BE = E_(complex)_ − E_(monolayer)_ − E_(NH3)_ (1)

In this equation, E_(complex)_ is the energy of the NH_3_ adsorbed VSe_2_/VSe_2_(V_v_)/VSe_2_(Se_v_) systems. The E_(monolayer)_ represents the energy of the VSe_2_ or VSe_2_(V_v_) or VSe_2_(Se_v_) systems. The last term E_(NH3)_ represents the energy of the isolated ammonia gas molecule.

The adsorption energy values are shown in Table 1. It can be observed from Table 1 that the NH_3_ molecule is weakly bound to the pure VSe_2_. Or, in other words, the NH_3_ shows weak affinity towards the VSe_2_ monolayer, specifying that pure material is not much suitable for sensing purposes. The result shown in Table 1 for the VSe_2_ + NH_3_ system corresponds to the adsorption energy of 0.124 eV for the case when the N atom of NH_3_ has been placed upright the V atom of VSe_2_. The same practice has been repeated for the other three possible sites, i.e., Se atom, V-Se bond, and center of a hexagonal ring consisting of V and Se atoms, and all four obtained adsorption energy values are shown in Table S1. As can be seen, the adsorption energy for the arrangement corresponding to the “above V” case is the least (though positive without VdW incorporation); further, all calculations are based on that arrangement. However, VSe_2_(V_v_) and VSe_2_(Se_v_) monolayers show stronger affinity towards NH_3_ with adsorption energy values of −0.22 and −0.66 eV, respectively. The present studies also determined the influence of long-range interactions by computing the adsorption energy values with DFT-D3 functional to consider van der Waal interaction. It can be observed from Table 1 that the adsorption energy values improve with the inclusion of VdW interactions. The values reported in Table 1 suggest that the VSe_2_(Sev) + NH_3_ forms the most stable complex due to higher adsorption energy values. The bond lengths between NH_3_ and the adsorbent are also measured and are given in Table 1. In the case of VSe_2_(Sev) + NH_3_, the distance between the vanadium atom of the monolayer and the N atom of NH_3_ is reduced as compared to the VSe_2_ + NH_3_ complex. This supports stronger adsorption interactions between VSe_2_(Se_v_) and the NH_3_ molecule. As the VSe_2_(Se_v_) + NH_3_ forms the most stable complex, the change in the electronic properties of pure and Se-defected monolayers with the adsorption of NH_3_ molecule is studied in this work and has been comparatively discussed further.

**Figure 4:**
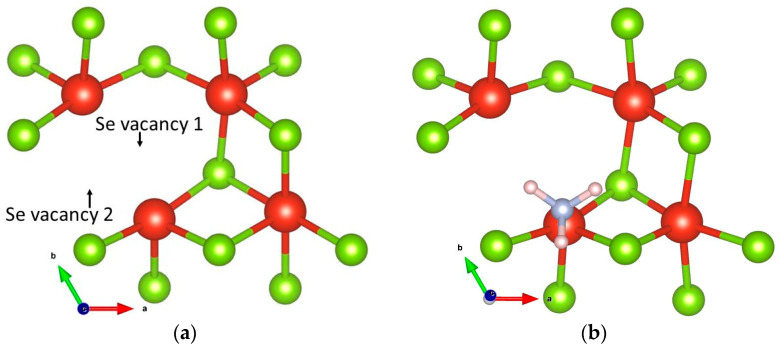
Relaxed structures of (**a**) VSe_2_ deficient with 2 Se atoms and (**b**) NH_3_ (N atom directly placed above V atom) on VSe_2_ deficient with 2 Se atoms.

To study the effect of a further increase in defect density, a VSe_2_ structure deficient with two Se atoms has been relaxed and again optimized with the insertion of an NH_3_ molecule. (Figure 4). The resultant adsorption energy values (−1.33 and −1.58 eV with VdW), as shown in Table 1, indicate stronger adsorption. Such observation is promising to conclude that doubling the Se vacancy population is beneficial for better NH_3_ detection.

### 3.3. Total Density of States (TDOS) Plots

**Figure 5:**
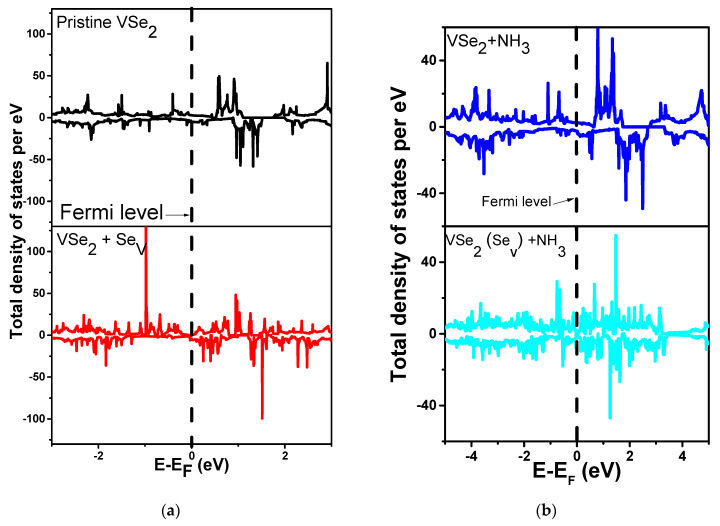
Comparison of TDOS plots between (**a**) Pristine VSe_2_ and VSe_2_ with Se Vacancy, and (**b**) NH_3_ adsorbed on pristine VSe_2_ on V atom, and NH_3_ adsorbed on VSe_2_ with Se vacancy on V atom.

In order to get insights regarding charge transfer and the interaction mechanism of NH_3_ with pristine and defected VSe_2_, we have presented total and partial density of states analyses. The TDOS plot of a pure VSe_2_ monolayer is specified in Figure 3a. To determine the magnetic behavior, spin-up and spin-down states are plotted. It is observed from the figure that the pure material is magnetic due to the asymmetry in spin states. The existence of the density of states at the fermi level implies the metallic behavior of the materials, consistent with earlier findings [64,65]. The total density of states enhanced by the adsorption of the NH_3_ molecule on VSe_2_ is shown in Figure 5a. In the case of the VSe_2_(Se_v_) system, an enhancement in TDOS is observed below the Fermi level, as depicted in Figure 5b. The enhancement in the density of states occurs due to the unbound V-atom bonds after the removal of the Se atom from the monolayer. The change in the density of states with the adsorption of NH_3_ supports the orbital interactions. The density of states is also enhanced at the fermi level with the adsorption of NH_3_ on the VSe_2_(Se_v_) system.

### 3.4. Partial Density of States (PDOS) Plots

**Figure 6:**
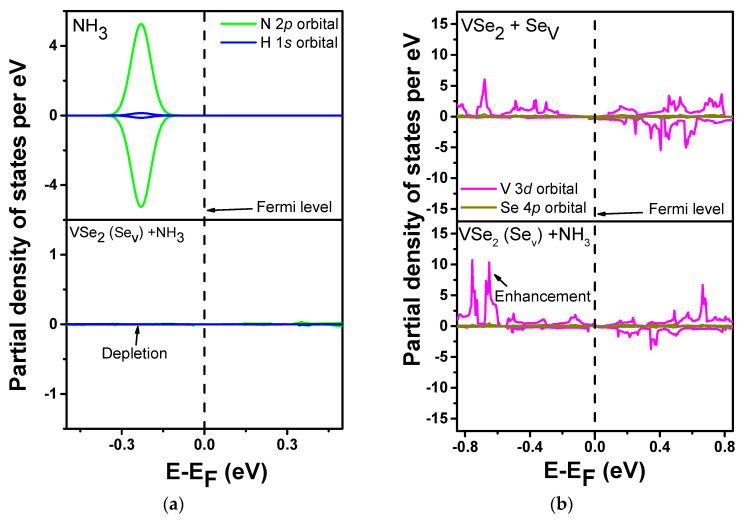
PDOS plots for (**a**) N 2p and H 1s orbital in NH_3_ and NH_3_+ VSe_2_ with Se vacancy and (**b**) V 3d and Se 4p orbital in VSe_2_ with Se vacancy and NH_3_+ VSe_2_ with Se vacancy.

To investigate the orbital interactions, the spin-polarized partial density of states (PDOS) is analyzed. The spin-polarized partial density of states (PDOS) for N-2p and H-1s orbitals in NH_3_ and VSe_2_(Se_v_) + NH_3_ were computed and are shown in Figure 6a. In the case of the NH_3_ molecule, the partial density of states for N-2p and H-1s orbitals is spotted in the valence band. These partial densities of states disappeared (or were reduced) with the adsorption of NH_3_ on the VSe_2_(Se_v_) monolayer. Further, the spin-polarized partial density of states (PDOS) of V-3d orbitals for VSe_2_ + Se_v_ and VSe_2_(Se_v_) + NH_3_ were computed and are shown in Figure 6b. On comparing the PDOS of V-3d orbitals of VSe_2_(Se_v_) and VSe_2_(Se_v_) + NH_3_ systems, it can be observed that the densities of states are enhanced in the latter with the adsorption of the NH_3_ molecule. This suggests that the monolayer is acting as an electron acceptor, whereas NH_3_ is acting as an electron donor. So, we can say that there is a charge transfer from NH_3_ to VSe_2_(Se_v_) due to the adsorption of NH_3_.

The total density of states and partial density of states plots have shown that the electronic properties of the VSe_2_ monolayer can be tuned with the defect induction, which impacts the adsorption properties.

### 3.5. Charge Transfer Analysis

**Figure 7:**
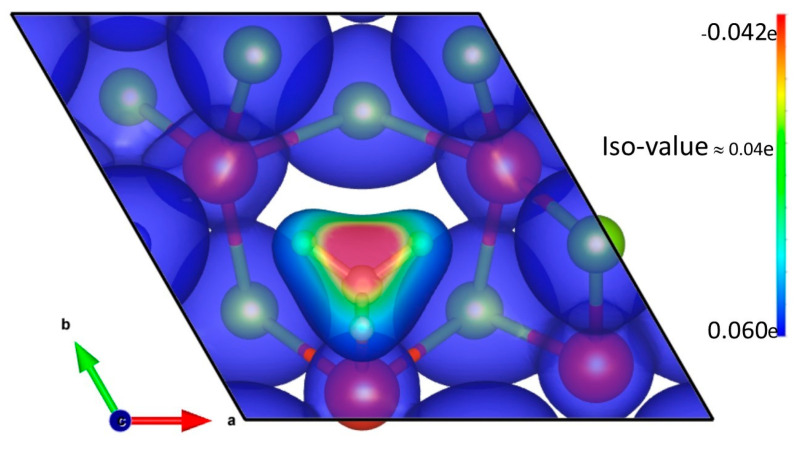
The charge density difference plot of NH_3_-attached Se-deficient VSe_2_. Red regions indicate charge loss, whereas blue and green regions indicate charge gain.

The interactions between the analyte and host were determined in terms of Bader charge analysis [82]. The VSe_2_(Se_v_) monolayer shows a net gain of 0.009e of charge due to adsorption of the NH_3_ molecule whereas, the NH_3_ molecule shows a net loss of 0.009e of charge, suggesting that the monolayer acts as an electron acceptor. The Bader charge analysis is in accordance with the partial density of states (PDOS) plots (Figure 6). The above observation is consistent with the opinion of earlier researchers regarding ammonia sensing in terms of charge transfer course. (Table 2) [37,83,84,85,86]. Additionally, a charge density difference plot has been shown in Figure 7. It is performed with the relation:

$$\rho_{Difference}=\rho_{VSe_{2}\left(Se_{V}\right)+NH_{3}}-\rho_{VSe_{2}\left(Se_{V}\right)}-\rho_{NH_{3}}$$

For all three systems, the ISO values are around 0.04e, wherein red regions denote regions of charge loss and green or blue regions denote charge gain. In all three systems, a charge loss region is noted around the N atom of the NH_3_ molecule, while a charge gain region is noted over the VSe_2_ surface with a Se vacancy.

### 3.6. Thermal Stability from Molecular Dynamics Simulations

**Figure 8:**
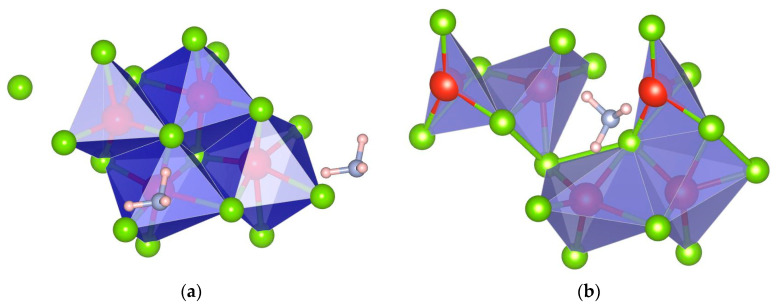
MD snapshots (**a**) VSe_2_ + NH_3_ (**b**) VSe_2_(Se_v_) + NH_3_ at 400 K after 5 ps; for the pristine VSe_2_: as adsorption energy is less, NH_3_ is desorbed while for VSe_2_ (Se_v_) it remains intact.

A nanosensor should be stable at higher temperatures for its efficient performance. Moreover, the gas molecules adsorbed on it should remain intact in the system until the sensing procedure is completed. As pristine VSe_2_ is a synthesized material, it is thermally stable at room temperature. So, we have investigated the thermal stability of VSe_2_ + NH_3_ and VSe_2_(Se_v_) + NH_3_ systems. The ab initio molecular dynamics simulations were carried out to investigate the thermal stability of the considered material at higher temperatures. The snapshots of equilibrated VSe_2_ + NH_3_ and VSe_2_(Se_v_) + NH_3_ systems after 5 ps at 400 K are shown in Figure 8.

**Figure 9:**
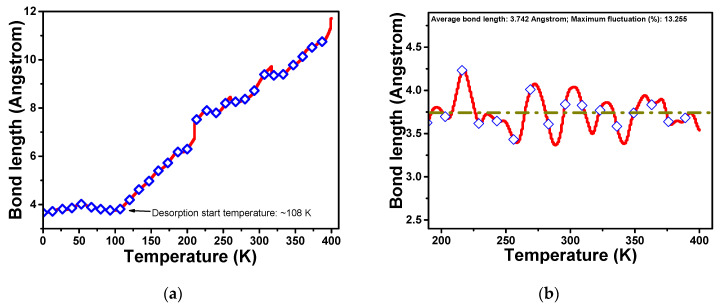
Variation of bond length N-V with the temperature during AIMD simulations for (**a**) VSe_2_ + NH_3_ (**b**) VSe_2_(Se_v_) + NH_3_; for pristine VSe_2_, NH_3_ is desorbed while for VSe_2_(Se_v_) it remains intact.

The bond length fluctuations (between N of NH_3_ and V of VSe_2_) with the temperature are plotted in Figure 9. We can notice that for pristine VSe_2_, the NH_3_ molecule goes away from the system starting with a temperature of 108 K. It seems that the NH_3_ molecule desorbs from the system once the temperature is increased, with desorption starting around 108 K. This is because NH_3_ is bonded very weakly on pristine VSe_2_ and goes out of the system at higher temperatures. So NH_3_ desorbs from the system below room temperature for pristine VSe_2_. So, pristine VSe_2_ is not suitable for NH_3_ sensing due to weaker interactions and low adsorption energy. But for VSe_2_(Se_v_) + NH_3_ system, the bond length fluctuations are not much. It is around 10% of the mean value, suggesting that adsorbed NH_3_ remains intact at 300 K and even up to 400 K on the sensing material. This is due to the fact that the adsorption energy of NH_3_ on defected VSe_2_ has increased from −0.12 eV for the pristine system to −0.97 eV for the VSe_2_(Se_v_) system. Strong adsorption energy is due to charge transfer from NH_3_ to defected VSe_2_. So, the defected VSe_2_ is promising for NH_3_ sensing.

### 3.7. Recovery time (τ)

The reversible sensors could be used repeatedly and hence, are economically convenient for utilization in industrial sectors [65]. The recovery time analysis helps to determine the extent to which a sensor can be used reversibly. The recovery time determines the time required for an analyte to desorb from the host surface. It can be computed using the following equation [65]:

τ = ν^−1^exp(−E_ads_/kT) (2)

In the equation, the ν denotes the frequency factor or the reciprocal of the pre-exponential factor of the Arrhenius equation [87]. The terms E_ads_, k, and T denote the adsorption energy, Boltzmann constant, and temperature, respectively.

Using this equation, the recovery time for VSe_2_ + NH_3_ and VSe_2_(Se_v_) + NH_3_ systems were computed at 300 K and 500 K for visible yellow light and UV light. The τ values are shown in Table 3. The tabulated values show that at 300 K under UV radiation, VSe_2_(Se_v_) + NH_3_ system promises a convenient recovery time (~2 s). This suggests that VSe_2_(Se_v_) + NH_3_ system can act as a reusable sensor.

Apart from the above, response time is also considered a very important parameter for determining the sensitivity of any gas detector. When the gas is initially applied, it takes a few seconds for the sensor output current to attain steady-state conditions [88]. The response time of the sensor is commonly specified by the T_90_ or T_50_ time. T_90_ is the time for the sensor’s response current to reach 90% of its steady-state value. Similarly, the T_50_ metric is the time required for the sensor to reach 50% of its steady-state value [88]. Future progress in this work can consist of determining the response time for VSe_2_ to detect NH_3_.

In spite of promising results, improvements in 2D VSe_2_ are needed to attain better sensitivity, selectivity, and stability. Specifically, there is scope for improvement in recovery time owing to the slow gas desorption process to enable it suitable for usage at room temperature. Currently, this kind of resource seems to be substandard in terms of sensing presentations when contrasted with metal oxide nanostructures; however, their performance is on par with that of pristine graphene. The technology available as of now to physically fabricate planer structures is still not industrially budget-friendly, so more technological advancement is necessary.

## 4. Conclusions

The structural, electronic, and sensing properties of pure and defected VSe_2_ monolayers have been investigated with density functional theory calculations. The energetic stability of VSe_2_(Se_v_) + NH_3_ and VSe_2_(V_v_) + NH_3_ monolayers is studied as adsorption energy values. The VSe_2_(Se_v_) binds strongly with the adsorbed NH_3_ molecule compared to the pure nanomaterial. With the introduction of Se vacancy, the adsorption energy increases from −0.12 eV in the pristine case to −0.97 eV for VSe_2_(Se_v_). Charge transfer from NH_3_ to defected VSe_2_ is responsible for stronger adsorption. It has been observed that NH_3_ acts as a charge donor and the host, i.e., VSe_2_, as a charge acceptor to cause the adsorption to be effective. The thermal stability of the VSe_2_(Se_v_) + NH_3_ system was investigated by performing ab initio molecular dynamics simulations at 300 K and 400 K and the system was found to be structurally stable even at higher temperatures. The recovery time analysis suggests that the VSe_2_(Se_v_) monolayer can act as a reusable nanosensor. The present studies show that the sensing properties of the VSe_2_ monolayer can be significantly improved with the introduction of Se-defects in the lattice structure. Or, in other words, tuning structural and electronic properties through the introduction of Se vacancy aids in enhancing the sensing properties of the VSe_2_ monolayer for NH_3_ adsorption. The obtained results will be potentially helpful for experimentalists to design defect-engineered TMD-based novel gas sensors.

## Figures and Tables

**Table 1:** Adsorption energies for the adsorption of NH_3_ on VSe_2_, VSe_2_(V_v_), and VSe_2_(Se_v_) systems with and without VdW functional. The bond lengths between the atoms of adsorbate and adsorbent are given in Å units.

System	Adsorption Energy (eV)	Bond Length (Å)
VSe_2_ + NH_3_	0.124	V-N: 4.786 S-N: 3.94
VSe_2_ + NH_3_ (with VdW)	−0.12	V-N: 4.709 S-N: 3.93
VSe_2_ (V vacancy) + NH_3_	−0.219	V-N: 4.756 S-N: 3.92
VSe_2_ (V vacancy) + NH_3_ (with VdW)	−0.342	V-N: 4.479 S-N: 3.732
VSe_2_ (Se vacancy) + NH_3_	−0.664	V-N: 2.26 S-N: 3.697
VSe_2_ (Se vacancy) + NH_3_ (with VdW)	−0.97	V-N: 2.253 S-N: 3.681
VSe_2_ (2Se vacancy) + NH_3_	−1.33	V-N: 2.242 S-N: 3.514
VSe_2_ (2Se vacancy) + NH_3_ (with VdW)	−1.58	V-N: 2.241 S-N: 3.501

**Table 2:** Comparison with earlier reported charge transfer data for NH_3_ sensing.

2D Material	Charge Lost by NH_3_	Reference
MoS_2_/ WS_2_	0.09e/0.03e	[37]
Ag_3_-WSe_2_ monolayer	0.202e	[83]
MoS_2_	Pictorial illustration	[84]
Ti_3_C_2_T_x_ MXene @ TiO_2_/MoS_2_ heterostructure	~0.03e	[85]
WOS nanosheet	Pictorial illustration	[86]
VSe_2_(Se_v_)	0.009e	This work

**Table 3:** Recovery time for VSe_2_ + NH_3_ and VSe_2_(Se_v_) + NH_3_ systems at 300 K and 500 K for yellow light and UV light.

System	Recovery Time (s)			
	Yellow Light (ν = ~5.2 × 10^14^ Hz)		UV Radiation (ν = 1 × 10^14^ Hz)	
	300 K	500 K	300 K	500 K
VSe_2_ + NH_3_ (with VdW)	1.97 × 10^−13^	3.09 × 10^−14^	1.02 × 10^−14^	1.61 × 10^−15^
VSe_2_(Se_v_) + NH_3_ (with VdW)	1.92 × 10^−15^	1.16 × 10^−05^	*1.99*	6.01 × 10^−07^

## Data Availability

The data presented in this study are available on reasonable request to the corresponding author.

## Supplementary Materials

The following supporting information can be downloaded at: https://www.mdpi.com/article/10.3390/bios13020257/s1, Table S1: Adsorption energies for the adsorption of NH3 on VSe2 at different sites.
