# Supplementary material for: Defect-Engineering of 2D Dichalcogenide VSe2 to Enhance Ammonia Sensing: Acumens from DFT Calculations
Source: Biosensors (Basel). 2023 Feb 11;13(2):257. doi: 10.3390/bios13020257 (PMC9954586; doi:10.3390/bios13020257)
Supplement: Supplementary file 1 [file biosensors-13-00257-s001.zip › biosensors-2108839-supplementary.pdf]

Defect-engineering of 2D dichalcogenide VSe_2 to enhance ammonia sensing: Acumens from DFT Calculations

Gopal Sanyal[1], Surinder Pal Kaur[2], Chandra Sekhar Rout[3*], Brahmananda Chakraborty[4,5*]

1 Mechanical Metallurgy Division, Bhabha Atomic Research Centre, Trombay, Mumbai 400085, India
2 Department of Chemistry, Indian Institute of Technology Ropar, Rupnagar 140001, India
3 Centre for Nano and Material Sciences, Jain Global Campus, Jakkasandra, Ramanagaram, Bangalore 562112, India
4 High Pressure and Synchroton Radiation Physics Division, Bhabha Atomic Research Centre, Trombay, Mumbai 400085, India
5 Homi Bhabha National Institute, Mumbai 400094, India
* Correspondence: brahma@barc.gov.in

Supporting information

Table S1. Adsorption energies for the adsorption of NH_3 on VSe_2 at different sites

**System**	**Site for placing N atom of NH_3 on VSe_2 above 2 Å from surface**	**Adsorption energy (eV)**
VSe_2+NH_3	V atom	0.124
VSe_2+NH_3	Se atom	0.135
VSe_2+NH_3	V-Se bond	0.218
VSe_2+NH_3	Centre of hexagonal ring consisting of V and Se atoms	0.147
